# Biological functions of 5-methylcytosine RNA-binding proteins and their potential mechanisms in human cancers

**DOI:** 10.3389/fonc.2025.1534948

**Published:** 2025-02-07

**Authors:** Tingting Zhao, Zhe Zhang, Zhuo Chen, Guozheng Xu, Yongxi Wang, Fang Wang

**Affiliations:** Department of Breast Surgery, The First Affiliated Hospital of Zhengzhou University, Zhengzhou, China

**Keywords:** 5-methylcytosine, m5C-binding proteins, detection techniques, biological functions, tumor regulation

## Abstract

The 5-methylcytosine (m5C) modification is a crucial epigenetic RNA modification, which is involved in the post-transcriptional regulation of genes. It plays an important role in various biological processes, including cell metabolism, growth, apoptosis, and tumorigenesis. By affecting the proliferation, migration, invasion, and drug sensitivity of tumor cells, m5C methylation modification plays a vital part in the initiation and progression of tumors and is closely associated with the poor tumor prognosis. m5C-related proteins are categorized into three functional groups: m5C methyltransferases (m5C writers), m5C demethylases (m5C erasers), and m5C methyl-binding proteins (m5C readers). This paper introduces several common methodologies for detecting m5C methylation; and reviews the molecular structure and biological functions of m5C readers, including ALYREF, YBX1, YBX2, RAD52, YTHDF2, FMRP, and SRSF2. It further summarizes their roles and regulatory mechanisms in tumors, offering novel targets and insights for tumor treatment.

## 1 Introduction

Research on the mechanisms of RNA regulation in tumors has increased during the last five years. Epigenetic regulation of RNAs represents an important aspect of RNA regulation, influencing the expression of mRNAs, tRNAs, rRNAs, and other non-coding RNAs at the post-transcriptional level (1, 2). The m5C methylation modification is one of the most common RNA modifications, which is associated with gene expression and stability (3). m5C modification has been found to promote tumor progression and associate with poor prognosis in several tumor types, including hepatocellular carcinoma, pancreatic cancer, esophageal cancer, and breast cancer (4–7).

**Figure 1:**
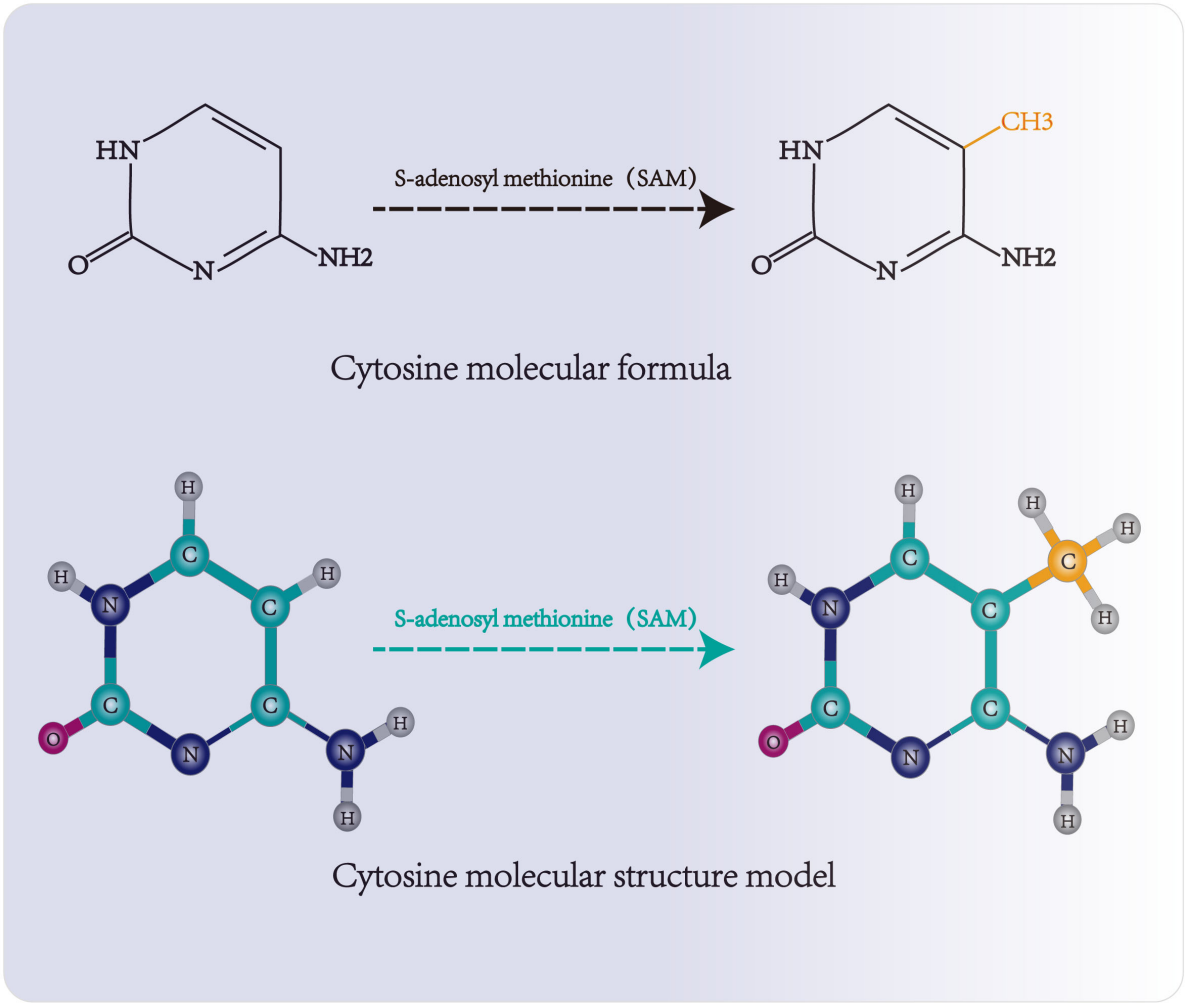
Molecular structure and methylation site of cytosine. Methyltransferase transfers the methyl group of S-adenosylmethionine to the fifth carbon atom of cytosine, forming 5-methylcytosine.

**Figure 2:**
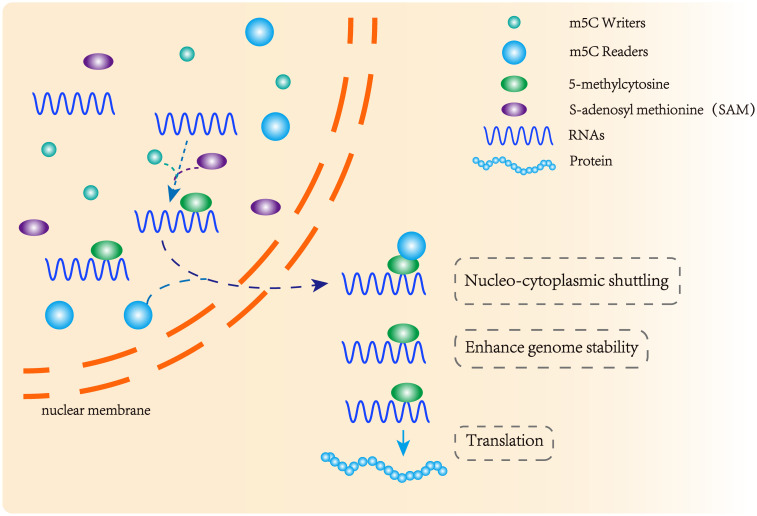
m5C methyltransferase and m5C binding protein participate in the formation of methylation modification. m5C methyltransferase catalyzes the formation of m5C methylation, and the methylation binding protein recognizes methylated mRNA, promoting its nuclear transport and affecting its stability and post-transcriptional translation.

**Figure 3:**
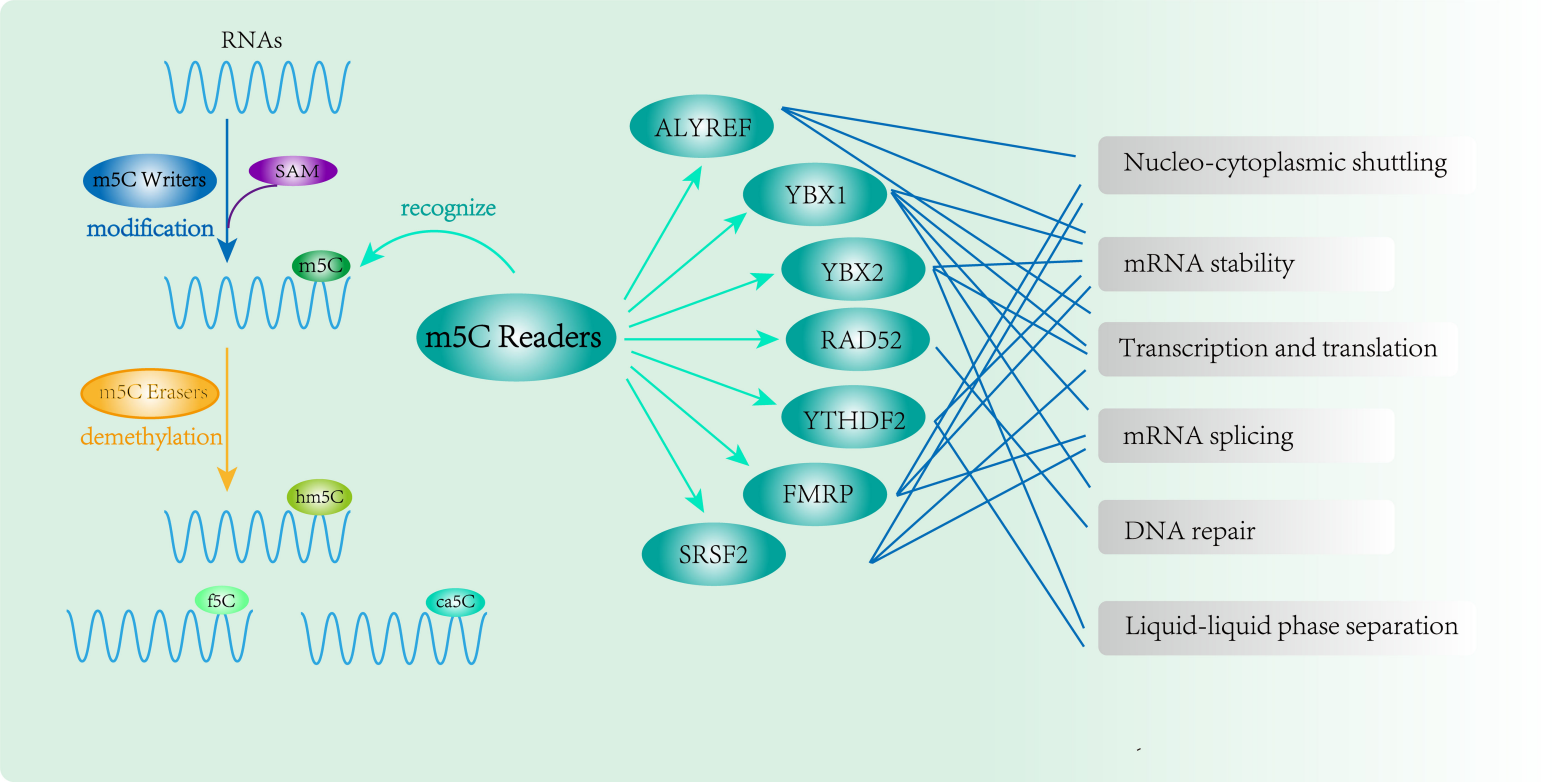
RNA m5C modification and function of m5C Readers. m5C binding proteins(ALYREF, YBX1, YBX2, RAD52, YTHDF2, FMRP and SRSF2) participate in methylation modification and perform complex epigenetic regulation of genes.

Three functional components are necessary for m5C modification and gene regulation. Firstly, the methyltransferase transfers the methyl group from S-adenosylmethionine to the fifth carbon atom of cytosine, thereby forming the m5C modification (8) (**Figure 1**). Secondly, methyl-binding proteins or demethylases identify and bind methylated mRNA, which in turn affects biological behavior, realizes epigenetic regulation of genes, involves metabolism and tumorigenesis in the human body (9) (**Figures 2**, **3**, **Table 1**). In addition to gene regulation, Ding et al. found a link between m5C methylation sites in hepatitis B patients and the virus’s ability to replicate and evade antiviral treatments (10). Additionally, it has been demonstrated to influence adipogenesis by regulating the cell cycle and autophagy (11). Aberrant methylation of m5C leads to malignant proliferation of gastric cancer cells and poor prognosis by promoting reprogramming of glutamine metabolism (12). It can be seen that m5C methylation modification not only leads to malignant outcomes such as tumors but also participates in some fundamental life processes of cells.

**Table 1:** Summary of RNA m^5^C readers.

Regulator	Structural features	Cellular distribution	Biological processes involved	Identifiable methylation types
ALYREF	*-*	Nucleus	Specifically recognizes and binds m5C mRNAs, mediates nucleo-cytoplasmic shuttling, transcription elongation, genome stability, the chaperone of basic leucine zipper (bZIP)	m5C
YBX1	A cold-shock domain	Cytoplasm	RNA stabilization, mRNA splicing, DNA repair, translational and transcription regulation, cell migration and proliferation	m5C
YBX2	A cold-shock domain	Nucleus	Major constituent of messenger ribonucleoprotein particles (mRNPs), regulate the stability and translation of germ cell mRNAs, liquid-liquid phase separation	m5C
RAD52	A heptameric circular DNA-binding protein resembling a windmill	Nucleus	Genetic recombination, DNA repair	m5C
YTHDF2	Three aromatic amino acid residues in the hydrophobic pocket	Nucleus and Cytoplasm	Regulate mRNA stability, regulate cell growth and cell cycle, regulate immunity, regulates circadian regulation of hepatic lipid metabolism, liquid-liquid phase separation	m5C, m6A, m1A
FMRP	Three structural domains: the N-terminus, the central structure, and the C-terminus	Nucleus and Cytoplasm	Neuronal development and synaptic plasticity, mRNA nuclear export, alternative mRNA splicing, mRNA stability	m5C, m6A
SRSF2	Rich in serine and arginine residues	Nucleus and Cytoplasm	Alternative mRNA splicing, mRNA nuclear export, transcription and translation, immune depletion	m5C

Although m5C methyltransferases have been thoroughly studied, the members of the m5C methyl-binding protein family have not yet been systematically elucidated. In this review, we summarize the m5C Readers identified in the literature as comprehensively as possible, refine and update the m5C methyl-binding protein members, and primarily describe the molecular structure characteristics, biological functions and mechanisms of m5C methyl-binding proteins (ALYREF, YBX1, YBX2, RAD52, YTHDF2, FMRP and SRSF2) in tumorigenesis and development.

## 2 Regulators of m5C methylation

The regulators of m5C modification can be divided into three groups according to their functional characteristics, including m5C methyltransferases (m5C writers), m5C demethylase (m5C erasers), and m5C methyl-binding proteins (m5C readers).

The m5C writers primarily consist of the NSUN family (NSUN1-NSUN7) and the methyltransferase homologue TRDMT1 (DNMT2) (8, 13, 14), which form m5C methylation by transferring the methyl group to the fifth carbon atom of cytosine. m5C methyltransferases are involved in the formation of methylation, modulate RNA function and stability, influence post-transcriptional modifications, and are involved in tumorigenesis and progression (8).

The m5C erasers including ALKBH1 and TET families (15, 16), which is capable of oxidizing m5C, thereby achieving the effect of removing methyl groups from methylated RNA, that is to say, the demethylation of the RNA. Similarly, ALKBH1 and TET play a pivotal role in tumorigenesis by influencing methylation formation and regulating the malignant phenotype of cancer cells, which is closely relevant to poor prognosis (17, 18).

The m5C readers include ALYREF, YBX1, YBX2, RAD52, YTHDF2, FMRP and SRSF2 (19–25). They can specifically recognize m5C-methylated RNAs, affect RNA stability and nucleocytoplasmic shuttling, and regulate transcription and translation. At the cellular level, they can enhance the ability of proliferation, migration, and invasion of cancer cells. They are also correlated with the immune microenvironment and drug resistance of tumors, which can further accelerate the tumor progression (26, 27).

The different acting elements of m5C work together to promote the occurrence and development of tumors. For example, the co-expression of ALYREF and NSUN2 is frequently observed in bladder cancer, and both proteins regulate RNA methylation and post-transcriptional modifications to promote bladder cancer progression (28). YBX2 and YTHDF2 interactions promote the stability of mRNA of endometrial cancer cells (29). In non-small cell lung cancer, the combined action of NSUN2 and YBX1 results in the increased expression of the target gene *QSOX1*, which mediates resistance to gefitinib in non-small cell lung cancer (30). NSUN 2 and YBX 1 are closely related, and in addition to non-small cell lung cancer, they can also interact in pancreatic, colorectal cancer, and ovarian cancer to jointly regulate tumor progression (4, 31, 32).

## 3 Detection methods of m5C methylation modification

Epigenetic modification of gene has increasingly become the focus of research, and the excellent performance of m5C methylation modification of RNA in the development of tumors has attracted more and more researchers. This also means that we need more convenient and efficient methods to help researchers detect the presence or extent of methylation. With the rapid development of scientific testing technology, more and more methods can be chosen to detect the existence, abundance, or type of m5C methylation, which can be selected according to the purposes of the detection, and here are some common methods.

### 3.1 Immuno-northern blotting

RNA obtained by electrophoretic separation was transferred to a PVDF membrane and incubated with an antibody specific for the m5C methylation modification, followed by a chemiluminescent reaction to show the location of the bands to assess if the m5C methylation modification is present (33).

### 3.2 The second generation sequencing

The second generation sequencing, a transcriptomic detection method, has been the preferred approach for RNA detection due to its low error and high accuracy. The examination of m6A and m5C alterations commonly uses nanopore technology, an amplification-free sequencing technique. Structural changes that occur as RNA or DNA passes through the nanopore result in differences in current blocking, which is translated into base sequences by a recurrent neural network (RNN) as a way of analyzing molecular signatures and locating regions of RNA methylation (34, 35).

### 3.3 Enzyme-linked immunosorbent assay

In order to determine the degree of methylation in the test samples, the signal intensities of the sample RNAs are compared to standard curves obtained from known methylated and non-methylated control RNAs (34). Commercially available kits are sufficient for the assay.

### 3.4 Mass spectrometry

RNA is broken down into nucleosides by enzymatic digestion, and these are then purified. Mass spectrometry is then used to separate the ribonucleosides and identify the methylated modified nucleosides. Individual molecules are identified and measured according to their mass-to-charge ratio (36). The method takes advantage of the fact that the modified nucleotide differs from its unmodified counterpart in terms of net charge, hydrophobicity, and polarity (34). The classical methods for the analysis of RNA methylation modification are one-dimensional (1D) or two-dimensional (2 D) thin-layer chromatography (TLC) and liquid chromatography-tandem mass spectrometry (LC-MS/MS) (36).

### 3.5 Hybridization with spots

Spot hybridization, also known as slit hybridization, is a relatively simple method for the detection of methylation patterns in diverse RNA types. The membranes with RNA are incubated with specific antibodies or probes to determine the existence and level of methylation by the depth of the developed spots (37). This experiment should only be used to verify the presence of methylation and to compare the change in the overall abundance of methylation between different samples. However, it is equally significant to be careful to avoid contamination of the DNA, as this may affect the results.

## 4 Molecular structure and biological function of m5C RNA-binding proteins

### 4.1 ALYREF

ALYREF, also designated THOC4, is a heat-stable nuclear protein that has the unusual ability to recognize m5C sequences and act as a molecular chaperone. ALYREF is known to facilitate the dimerization of unfolded leucine zips by recognizing them, to activate transcription. It can also bind to specific molecules in the translational region of mRNAs, thereby regulating gene expression, mRNA egress from the nucleus and genome stability (38–42). In most tumors, ALYREF has a high expression level and is closely associated with tumor heterogeneity, immune infiltration, and a high mutation rate of *TP53*, which enhances the proliferation, migration, and invasive properties of tumor cells, as well as drug resistance, tumor progression and adverse prognosis (43–45). The activity of ALYREF is regulated by AKT-mediated phosphorylation, with reduced ALYREF phosphorylation observed to suppress cell proliferation and mRNA export (46).

### 4.2 YBX1

YBX1 is a gene with a wide range of nucleic acid binding properties encoding a highly conserved cold-excited structural domain protein that plays a pivotal role in many basic biological functions, such as transcription, translation, DNA repair, and splicing of precursor mRNAs (47–49). YBX1 displaces translation initiation factors from messenger ribonucleic acid bodies and redistributes them to improve translation efficiency (50). However, the regulation of translation by YBX1 is not always positive. Studies have demonstrated a bell-curve relationship between the relative amount of YBX1 and the level of translation, with YBX1 acting as an inhibitor of translation when the relative amount of YBX1 is high (51–53). As a splicing regulator of messenger RNAs (mRNAs), YBX1 indirectly affects mRNA splicing and shear factors behavior by identifying particular sequences in precursor mRNAs (54). YBX1 can directly bind to m5C-methylated mRNAs and act to stabilize them (19, 20). In addition, YBX1 is essential for maintaining cardiomyocyte function. mTOR activation of YBX1 can transmit pathological signals and regulate protein synthesis in cardiomyocytes, which significantly contributes to the development of cardiac hypertrophy (55). YBX1, a known oncoprotein, shows high expression in a variety of tumors, and YBX1 can regulate tumor angiogenesis by releasing angiogenic factors into the extracellular microenvironment, playing a role as an oncogenic enhancer (56, 57).

### 4.3 YBX2

YBX2 is a member of the Y-box protein family, and similar to YBX1, YBX2 also has an alanine/proline (A/P)-rich N-terminal domain, a variable C-terminal domain (CTD), and a highly conserved cold shock domain (CSD) (58). YBX2 is a major component of mRNP and regulates mRNA stability and translation (29, 59, 60). Because MAPK phosphorylates YBX2, ubiquitination-mediated degradation is suppressed. The accumulated YBX2 then activates brown adipose tissue, thereby promoting glucose utilization and lactic acid production in glycolysis (61). In addition, methylated YBX 2 can be recognized by ALYREF, promoting YBX 2 nucleation and increasing YBX 2 protein expression, which is an important way to regulate lipolysis (62). Moreover, YBX2 is essential for maintaining the normal function of germ cells (63). m5C methylation regulates the activity of YBX2, a novel m5C binding protein, and that can undergo liquid-liquid phase separation *in vitro* and *in vivo* (21). Pan-cancer analysis showed that YBX family genes are associated with most tumor progression and can predict tumor prognosis to a certain extent (64).

### 4.4 RAD52

RAD52 is a multimeric cyclic DNA repair protein consisting of 418 amino acids. The human Rad52 protein, which is composed of a heptameric ring, resembles a windmill (65).In ROS-induced DNA double-strand break damage, RAD52 can preferentially bind to DNA: RNA hybrids containing m5C methylation-modified RNAs with the help of the m5C methyltransferase TRDMT1 to promote homologous recombination (23). This observation suggests that RAD52 may serve as a reader of m5C RNA in DNA: RNA hybrid at the DNA damage site and that m5C methylation modification of RNA is crucial for gene repair and maintaining genomic stability (66). Additionally, the activity, stability, and function of RAD52 are also affected by post-translational modifications such as phosphorylation and ubiquitination (67, 68). In tumors, RAD52 interacts with RNA polymerase-associated factor 1 (PAF1) to inhibit cisplatin and gemcitabine resistance (69).

### 4.5 YTHDF2

Based on structural and functional studies of YTHDF2, it was found to be a versatile reader that recognizes M6A, M1A and M5C (70, 71). Dai et al. demonstrated that three aromatic amino acid residues in the hydrophobic pocket of YTHDF2 that bind m6A can bind directly to m5C-methylated RNA. Considering that YTHDF2 recognizes multiple methylated forms, whether YTHDF2 acts on the m5C site can be verified by immunoprecipitation *in vitro* pull-down assays and LC-MS/MS, and can also be distinguished by the difference in YTHDF2 binding to the m5C and m6A docking structural domains (22). Functionally, YTHDF2 participates in the processing of precursor ribosomal RNAs (pre-rRNAs) in an m5C-dependent manner and regulates translation (22, 72). Histone lactation controls YTHDF2 expression, and tumors with high levels of YTHDF2 are associated with a poor prognosis. Additionally, YTHDF2 regulates immune processes by affecting immune cell infiltration in the tumor microenvironment (73, 74). The deletion of YTHDF2 in the tumor microenvironment has been observed to result in increased apoptosis and impaired suppression of Treg cells (75). Additionally, it has been shown to promote reprogramming of tumor-associated macrophages, enhance antigen cross-presentation, inhibit tumor growth, and enhance the efficacy of PDL-1 antibodies (76, 77). As YTHDF2 interacts with more than one type of methylation modification, the mechanism by which it acts needs to be further explored and differentiated. However, the role of YTHDF2 in tumor is still controversial, showing very different effects in different cancer types. For example, it plays a dual role in gastric cancer (78, 79). A comprehensive and detailed examination is necessary to clarify its precise mechanism of action.

### 4.6 FMRP

FMRP is an RNA-binding protein with three structural domains: the N-terminus, the central structure, and the C-terminus, which is associated with the structure and function of synapses, is involved in the exit of mRNA from the nucleus, and also has a wide range of regulation of gene expression (80, 81). Early nervous system development requires FMRP to identify and bind mRNAs in the hippocampus and cerebral cortex, which are critical for memory and learning (82). Furthermore, FMRP plays a role in RNA methylation modification as an m5C reader, facilitates the interaction between the methyltransferase TRDMT1 and the demethylase TET1, influences transcription and translation, and contributes to DNA damage repair and cell survival (24, 83). Beyond its role in gene regulation, FMRP is also important in metabolic processes. It has been demonstrated that FMRP deficiency increases hepatic protein synthesis and affects lipid metabolism, indicating that FMRP is involved in the regulation of systemic metabolic homeostasis (84). In solid tumors, FMRP up-regulation contributes to poor prognosis, inhibits immune attack, promotes tumor growth, immune escape and epithelial-mesenchymal transition (EMT) conversion (85, 86).

### 4.7 SRSF2

SRSF2 belongs to the family of RNA-binding proteins known as serine/arginine-rich (SR) proteins, which are involved in constitutive and selective splicing of RNAs and suppress intron splicing (87–89). SRSF2 contains an RNA recognition motif (RRM) and an RS domain, the former for binding to RNA and the latter for binding to other proteins. Additionally, the interactions between different SR splicing factors are realized in the RS domain (90). It has been reported that SR proteins play a role in the process of mRNA egress and translation, in addition to RNA splicing (91). Aberrant expression of SRSF2 is closely associated with tumorigenesis (92). Recent studies find that knockdown of NSUN2 decreases RNA methylation levels, reduces SRSF2 binding, and alters RNA splicing, which evidences SRSF2 can act as an m5C reader and may be associated with the development of malignancy (25, 93). Besides, SRSF2 has been linked to immune system depletion in the tumor microenvironment. It is a potential therapeutic target for reversing immune depletion because it regulates the transcription of immune checkpoint genes by influencing signal transduction and promoter recruitment (94). SRSF2 is a key molecule for cell survival and not only has a role in tumors but also regulates myocyte proliferation and myogenesis by preventing premature aging, differentiation, and apoptosis of myocytes (95). Moreover, SRSF2 acts as a strong transcriptional activator that promotes hepatic energy homeostasis and bile acid metabolism. Mutation or deletion in the expression of SRSF2 can lead to aberrant hepatic splicing, metabolic dysfunction, bile acid accumulation, and the subsequent induction of endoplasmic reticulum stress and oxidative stress, ultimately leading to liver failure (96).

## 5 The roles of m5C readers in human cancers

### 5.1 Hepatocellular carcinoma

Hepatocellular carcinoma (HCC) is the material cause of morbidity and mortality among all cancers, and is a major threat to global health (97). The majority of liver cancer patients are concentrated in Asia, with China having the most liver cancer cases (98, 99). The low survival rates of liver cancer creates a significant challenge for treating this disease (100, 101). As a binding protein for m5C methylation, ALYREF can stabilize RNA and activate subsequent signaling pathways to exert a tumorigenic effect (102). Xue et al. found that ALYREF deficiency can inhibit hepatocellular carcinoma cells proliferation and tumor growth, increase the rate of apoptosis, and is associated with tumor immune infiltration (103, 104). In hepatocellular carcinoma, the overexpression of YBX1 remodels the tumor microenvironment, increasing the infiltration of immune cells and the transcription of PD-L1 (105, 106). YBX1 interacts with circular RNA to promote metastasis and drug resistance of liver cancer: circASH2 influences the liquid-liquid phase separation and cytoskeletal remodeling of YBX1, thereby promoting the metastasis of hepatocellular carcinoma (107, 108); cFAM210A binds to YBX1, which reduces YBX1 phosphorylation and inhibits the trans-activating effect on EMT. At the same time, cFAM210A is regulated by YTHDF2, which induces cFAM210A degradation and promotes hepatocellular carcinoma progression (109). The elevated expression of RAD52 in hepatocellular carcinoma is linked to age and gender, and it is associated with promoting proliferation and migration of hepatocellular carcinoma cells (110). YTHDF2 is associated with stemness and drug resistance in hepatocellular carcinoma cells, improving genomic stability and promoting immune escape and angiogenesis, while also facilitating hepatocellular carcinoma metastasis *in vivo* (111–114). SRSF2 is frequently mutated or overexpressed in cancerous cells, and SRSF2 deletion can stimulate the regeneration of hepatic progenitor cells and the activation of oncogenes in hepatocellular carcinoma, which increases the proliferative and tumorigenic potential of hepatocellular carcinoma cells, mediates drug resistance, and promotes the progression of hepatocellular carcinoma (115, 116). FMRP is overexpressed in hepatocellular carcinoma, promotes the translation of STAT3, and can bind to STAT3 mRNA, thereby facilitating its localization to cellular protrusions and the promotion of hepatocellular carcinoma metastasis (117).

### 5.2 Pancreatic cancer

Pancreatic cancer is globally recognized as one of the deadliest malignant tumors, and clinical treatment is facing great challenges. There is an urgent demand to explore its pathogenesis, discover therapeutic targets, and prevent its progression more effectively (118). ALYREF can affect amino acid metabolism in pancreatic cancer cells, promote immune escape, and is associated with poor prognosis (119). YBX1 directly promotes mucin expression and establishes a barrier to prevent chymotrypsin from digesting pancreatic cancer cells, a mechanism that ensures the survival of pancreatic cancer cells in the pancreatic microenvironment (120). Overexpression of YBX1 in pancreatic cancer binds to the promoter of *GSK3β*, resulting in upregulation of *CBX3*, which activates TGF-β signaling to regulate the cell cycle and promotes the growth and proliferation of pancreatic cancer cells (121, 122). YBX1 also promotes IL-18 transcription, increases immune cell infiltration, and regulates the immune microenvironment in pancreatic cancer (123). YTHDF2 regulates EMT via YAP, which in turn hinders the migration and invasion of pancreatic cancer cells (124).

### 5.3 Esophageal carcinoma

Esophageal cancer belongs to malignant tumors of the digestive system and progresses rapidly in the later stages. As the tumor grows in size, it can nearly completely obstruct the esophagus, which greatly reduces patients’ quality of life. Research shows that esophageal cancer occurs more frequently in men, with higher morbidity and mortality rates compared to women (125). m5C, one of the most common RNA modifications, plays an important role in esophageal cancer progression. YBX1 is upregulated in most cancers and esophageal cancer is no exception. Both *in vivo* and *in vitro*, YBX1 promotes the proliferation, migration, and invasion of esophageal cancer cells (5). In esophageal carcinogenesis, long non-coding RNAs are significant (126). *LINC00941* can interact with YBX1, bind to the promoter region of *SOX2*, upregulate *SOX2* transcription, increase RNA stability, and promote the malignant phenotype of esophageal cancer (127). *MiR-323a-3p* inhibits the proliferation, migration, and invasion of esophageal cancer cells by regulating FMR1 (128).

### 5.4 Breast cancer

Breast cancer is the most prevalent malignant tumor and the second principal cause of cancer deaths in women (129, 130). ALYREF has been demonstrated to promote the development of breast cancer by affecting transcriptional regulation and mitochondrial energy metabolism, and regulate the growth, apoptosis, and migration of breast cancer cells (6). Jin et al. found that ALYREF, in addition to regulating the nuclear export of mRNA, also affects the stemness of breast cancer cells and is associated with adriamycin resistance (131). In breast cancer, YBX1 is linked to genetic stability; it can interact with m5C methyltransferase NSUN2 to influence mRNA stability, protein synthesis, and promote tumor progression (132–134). YBX1 regulates the invasion and migration of breast cancer cells by down-regulating the levels of the protein coronin-1C (135). The YBX1 protein has been demonstrated to regulate the proliferation of breast cancer cells through the PI3K/AKT/mTOR signaling pathway. Furthermore, there is evidence that YBX1 is connected to the development of tamoxifen resistance (136). The interaction between DSCAM-AS1 and YBX1 in positive feedback regulates the expression of ERα and promotes the progression of breast cancer (137). Wu et al. identified a new piRNA, named piR-YBX1. This piRNA can bind directly to YBX1, resulting in a reduction in the levels of both mRNA and protein. Additionally, YBX1 has been observed to bind to RAF1, an important role in the MAPK signaling pathway, which plays a crucial role in the development and progression of triple-negative breast cancer (138). RAD52 is connected to the breast cancer susceptibility genes BRCA1 and BRCA2. Research has shown that mutations in RAD52 can suppress certain BRCA2-related phenotypes in breast cancer (139, 140). In cells lacking BRCA2, overexpression of RAD52 can compensate for the loss of BRCA2-associated function. However, simultaneous deficiencies of both RAD52 and BRCA2 have been shown to be lethal to cells (141). YTHDF2 reverses RNA demethylase-induced alterations in cellular phenotype by increasing mRNA stability (72). Furthermore, it has been found to promote cell proliferation, invasion, and tumorigenic properties *in vitro*, as well as promoting osteolytic metastasis of breast cancer *in vivo* (142, 143). Triple-negative breast cancer is characterized by the absence of specific markers and therapeutic targets, which contributes to worse outcome and high recurrence and metastasis rates. The main treatment modalities for triple-negative breast cancer are chemotherapy and immunotherapy. YTHDF2 has been reported to affect the pre-tumor phenotypic polarization of macrophages and antigen-presenting signals between immune cells in triple-negative breast cancer. Furthermore, it also inhibits immune activity and associates with drug resistance (144). SRSF2, another m5C methylation binding protein, promotes angiogenesis under hypoxic conditions by selectively splicing vascular endothelial growth factor A (VEGFA) and is associated with poor prognosis in breast cancer (145). Similar to other tumors, FMRP expression is elevated in breast cancer. Furthermore, there are considerable variations in FMRP expression among metastatic breast cancer lesions, with low expression in the brain and bones and high expression in the liver and lungs. Consequently, FMRP is regarded as a prognostic factor for site-specific metastasis (146).

### 5.5 Lung cancer

Lung cancer has a high incidence rate in both men and women, and common types include lung adenocarcinoma, squamous cell carcinoma, and small cell lung cancer, of which adenocarcinoma and squamous cell carcinoma are collectively known as non-small cell lung cancer (NSCLC) (97, 147). A recent study has indicated that ALYREF and YTHDF2 are correlated with mRNA stability in lung adenocarcinoma and act on the YAP signaling pathway to alter immune cell infiltration in the tumor microenvironment (148, 149). Furthermore, they have been shown to enhance the secretion of exosomes and activate the downstream pathway in lung adenocarcinoma by regulating YAP transcription, which promotes drug resistance, tumor progression, and metastasis (150, 151). In lung adenocarcinoma, YBX1 is highly expressed and binds to the promoter region of *CDC25a* and *HOXC8*, thereby regulating cell cycle progression, cell proliferation, and apoptosis (152, 153). The *Runx3-miR-148a-3p* axis targets and regulates YBX1, adjusting the levels of multiple genes like Cyclin D1 and MMP2. This affects the proliferation, migration, and invasion of non-small cell lung cancer, and promotes NSCLC progression (154). `The regulation of YBX1 on the stemness of NSCLC is complex: on the one hand, it can inhibit the expression of MUC5AC and the integrin β4/pSrc/p53 signaling pathway, reducing lung cancer cell stemness and increasing the therapeutic sensitivity of erlotinib (155). On the other hand, YBX1 can promote the activation of NANOG to enhance the stemness and spheroidal ability of non-small cell lung cancer and regulates MUC1 transcription to promote cancer metastasis and stem cell properties (156, 157). The phase separation of YBX1 is identified as a key process in the development of non-small-cell lung cancer, affecting carcinogenesis and progression by regulating the biological behavior of cancer cells (158). RAD52 is expressed at high levels in non-small cell lung cancer, and regulates cell cycle and apoptosis and correlates with tumor size, degree of differentiation, lymphatic metastasis, and susceptibility (159, 160). SRSF2 is overexpressed in non-small cell lung cancer and interacts with long non-coding RNAs to up-regulate the expression of VEGFR1-i13, affecting the proliferation and invasion of lung cancer cells (161–163). In neuroendocrine lung tumors, SRSF2 also makes a difference. Highly expressed SRSF2 acts as a cell cycle regulatory protein that regulates the proliferation of lung cancer cells and promotes cancer progression (164).

### 5.6 Prostate cancer

Prostate cancer is a common malignant tumor in males, representing a substantial risk to the quality and longevity of life (165). Methylation modification of RNA has a visible impact on prostate cancer. The m5C-binding protein YBX1 inhibits ubiquitination of the androgen receptor, increasing intracellular androgen levels and stability. While AURKA phosphorylates residues of YBX1 and promotes its stabilization and nuclear translocation so that a positive feedback loop is formed and plays an important role in prostate cancer (166). YTHDF2 promotes mRNA stabilization and protein expression in androgen-negative prostate cancer, upregulates EMT-related factors, and activates the AKT pathway, which in turn promotes cell proliferation to promote metastasis (114, 167). Besides, A high level of SRSF2 expression is correlated with an adverse prognosis in prostate cancer (168).

### 5.7 Bladder cancer

Bladder cancer is a malignant cancers with a high recurrence and metastasis rates and has a poor prognosis (169, 170). Studies show that bladder cancer is strongly associated with genetic mutations and partly with epigenetic dysregulation (171, 172). In bladder cancer, ALYREF enhances the stability of mRNA encoding the glycolysis rate-limiting enzyme PKM2 in an m5C-dependent manner, upregulates PKM2 expression, and promotes the progression and deterioration of bladder cancer (173). ALYREF recognizes the NSUN2 locus on methylated mRNA and promotes mRNA stabilization to enhance the proliferation and invasion of bladder cancer cells in an m5C-dependent manner (28). The study revealed that YBX1 is crucial in bladder carcinogenesis and the tumorigenic effects of YBX1 were closely associated with glycolysis (19). Furthermore, the upregulation of glycolytic enzymes to facilitate glycolysis by regulating c-MYC and HIF1-α expression (56). SRSF2 interacts with miR-193a-3p, and compelling evidence suggests that this interaction is strongly associated with multidrug resistance in bladder cancer (174).

### 5.8 Colorectal cancer

Due to the unique disease characteristics of colorectal cancer, symptoms usually appear at a late stage, resulting in most cases being diagnosed late and treatment being passive. Research find that the morbidity of colorectal cancer is generally on the rise (175). Therefore, clarifying the pathogenesis of colorectal cancer will provide more possibilities for treatment. In colorectal cancer, the function of YBX1 is regulated by non-coding RNAs, transcription factor NF-κb, etc, and is associated with the activation of various signaling pathways (176, 177). *Lnc-SOX9-4* inhibits YBX1 degradation, stabilizes YBX1 protein levels, and accelerates the proliferation and metastasis of colorectal cancer cells (178). YBX1 is an m5C reader and can also be modified by methylation, and the modified YBX1 shows different effects in tumors: when methylated by PRMT5, YBX1 can inhibit the growth, migration, and invasion of colorectal cancer cells (176). Phosphorylated YBX1 activates the NF-κB signaling pathway and promotes colorectal cancer progression (179). The reprogramming of glucose metabolism by YBX1 and NSUN2 in an m5C-dependent manner promotes lactate production and accumulation. Furthermore, lactate accumulation positively feedback regulates the pro-cancer effects of NSUN2 by promoting NSUN2 transcription (32). YTHDF2 is widely involved in the pathogenesis of colorectal cancer, recognizing methylated XIST and mediating its degradation to inhibit colorectal cancer progression (180). YTHDF2 also plays a role in aerobic glycolysis in *P53* wild-type colorectal cancer and suppresses the malignant phenotype of the tumor (181). In terms of treatment, YTHDF2 increases the sensitivity of post-surgical radiotherapy in colorectal cancer patients (182). The expression of SRSF2 in colorectal cancer is apparently higher than that in normal tissues. The high expression of SRSF2 acts as a cell cycle regulator, promoting the proliferation of colorectal cancer cells both *in vivo* and *in vitro*, and contributing to the progression of colorectal cancer (92). Overexpression of FMRP and RAD52 is associated with colorectal cancer progression. FMRP regulates necrotic activation of cancer cells by controlling the expression of RIPK1 and results in poorer prognosis (66, 183).

### 5.9 Gastric cancer

Gastric cancer is one of the most common malignancies, with high malignancy and lethality. It is prone to metastasis, recurrence, and drug resistance, and is the second most common cause of cancer death (184). The study indicated that ALYREF has an influence on accelerating cell proliferation and metastasis by regulating the cell cycle and preventing cell apoptosis (185). Long non-coding RNAs (lncRNAs) have been reported to play a multitude of roles in cancer. One example is the *lncRNA PIN1P1*, which has a high expression in gastric cancer and promotes cancer progression by interacting with the YBX1 (186). Under hypoxic conditions, HIF-1 mediates high expression of YTHDF2, which is correlated with unfavorable prognosis of gastric cancer and increases the expression and stability of *CyclinD1*, promoting the proliferation of gastric cancer cells, and being associated with chemotherapy resistance (78). In contrast, the study conducted by Shen et al. reveals that YTHDF2 has the potential to impede the proliferation of gastric cancer cells by negatively modulating the *FOXC2* signaling pathway (79). It can be seen that YTHDF2 exercises multiple functions in gastric cancer, acting on different molecules or pathways and producing distinct regulatory effects on tumors. Acetylated SRSF2 promotes the methylation of precursor RNA in gastric cancer cells, stimulates cell proliferation and migration, and mediates the malignant phenotype of gastric cancer cells, which correlates with poor prognosis of gastric cancer (187).

### 5.10 Other tumors

YBX1 functions as a splicing factor, upregulating pro-carcinogenic VEGF165 to promote the proliferation, migration, and invasion of osteosarcoma cells and induce angiogenesis (188). YTHDF2 plays a role in osteosarcoma demethylation, inactivating the STAT3 pathway and inhibiting tumor cell proliferation, while simultaneously blocking the cell cycle and accelerating apoptosis (189). YBX1 is a key molecule in acute myeloid leukemia cell survival, regulating cell proliferation, apoptosis, cell cycle, and cell signal transduction (190). In ovarian cancer, high expression of YBX1 and YTHDF2 is relevant to poor prognosis, with YBX1 up-regulating the expression of E2F1 by a phase separation manner, leading to tumor progression (31, 191). In addition, YBX1 is closely related to drug resistance in ovarian cancer, improving the ability of cell gene damage repair by increasing the stability of mRNA, thereby enhancing the resistance of tumor cells to platinum-based chemotherapy drugs (192). YBX2 promotes the properties of endometrial cancer stem cells and enhances their pellet-forming ability and chemotherapy resistance (193). YBX1 inhibits apoptosis of renal clear cell carcinoma cells, promotes migration and invasion, and is a potential prognostic marker and therapeutic target for renal cell carcinoma (194). SRSF2 is not only involved in alternative splicing but also related to the expression of apoptotic genes. The expression of SRSF2 in renal clear cell carcinoma is lower, which inhibits the activity of caspase-9 and enhances the survival ability of renal cell carcinoma (195). m5C binding proteins YBX1, YBX2, and ALYREF are highly expressed in head and neck squamous cell carcinoma, accelerating cell proliferation and tumor metastasis, and are associated with poor prognosis (196–199).

## 3 Discussion

m5C methylation has been shown to contribute to the development of various diseases, and m5C methylation-binding proteins are vital elements of m5C methylation that have an indispensable impact on the function of m5C methylation. The increasing popularity of m5C methylation research will inevitably lead to a greater demand for m5C methylation detection to more intuitively identify the presence and type of methylation. This article comprehensively summarizes the structure, function, and common detection methods of m5C methyl-binding proteins that have been identified so far, and focuses on the impact these proteins have on the development and progression of various tumorigenesis, providing potential targets and new perspectives for the clinical treatment of tumors.

In addition to ALYREF and YBX1, this article complements the recently discovered m5C readers, enriching the understanding of m5C methylation. Current studies show that m5C methylation-binding proteins include ALYREF, YBX1, YBX2, RAD52, YTHDF2, FMRP, and SRSF2, which can perform functions independently or work together. m5C methylation-binding proteins can specifically recognize methylated genes, affect s, participate in RNA nucleation and other processes, and realize the regulation of gene expression at the post-transcriptional level. As important methylation forms, m6A and m5C are closely related, and some m5C methylation-binding proteins also act on m6A, such as YTHDF2, which can specifically recognize m5C and catalyze m6A. Understanding their interactions can help us to explore the synergies and networks between them, and benefit us to understand their mechanisms more comprehensively.

The m5C reader is associated with a variety of diseases, including various malignancies, and affects tumor proliferation, migration, invasion, and drug resistance by inducing cancer stem cell properties and promoting EMT transformation. In addition, methylation modification is related to tumor immunity. It regulates immune cell infiltration, changes the tumor immune microenvironment, mediates immune escape, and is closely associated with poor prognosis. Of course, there are two sides to everything, and m5C methylated binding proteins do not always contribute to promoting cancer, different binding proteins play varied roles in different tumors, and even the role of the same protein in the same cancer type is inevitably controversial. For example, high expression of YTHDF2 under hypoxic conditions promotes the proliferation and chemoresistance of gastric cancer cells (78). Similarly in gastric cancer, Shen et al. find that YTHDF2 can inhibit the growth of gastric cancer cells by negatively regulating the FOXC2 signaling pathway (79). Of course, current research on the mechanism of m5C methylation is still limited and one-sided and needs to be further explored and supplemented.

There is a rich variety of RNA modifications, and while abundant modification types have been discovered, other forms of modifications may still exist. Therefore, it is necessary to develop more precise and sensitive detection methods. For m5C methylation, in addition to the several detection methods described here, more specific strategies and innovative techniques are needed to detect RNA modification.

While it is essential to understand the molecular mechanisms of methylation, the ultimate goal of studying microstructure is to serve clinical treatment. Currently, there is still a gap in targeted drugs for m5C methylated molecules, and specific targeted inhibitors are worth further exploring. In addition, m5C methylation is associated with tumor immune invasion and can mediate drug resistance, suggesting that the use of targeted inhibitors may be expected to improve the effectiveness of immunotherapy and improve tumor prognosis. However, targeted RNA modification still faces great challenges, and it is necessary to overcome the difficulties of off-target and specificity, minimize the impact on unmethylated RNA, reduce side effects, and maximize the efficacy as much as possible. Secondly, RNA modification is a dynamic process, and achieving precise targeting of the modification site requires advanced technology. In a word, although the mechanism of action of m5C methylation-binding protein in tumors is controversial, various studies have shown that it is still a potential target for tumor treatment, providing new ideas for tumor treatment and bringing new hope to cancer patients.

## Glossary

m5C: 5-Methylcytosine
ALYREF: RNA and export factor-binding protein 2
YBX1: Y-box-binding protein 1
YBX2: Y-box-binding protein 2
RAD52: DNA repair protein
YTHDF2: YTH N6-methyladenosine RNA binding protein F2
FMRP: Fragile X messenger ribonucleoprotein 1
SRSF2: Serine and arginine-rich splicing factor 2
NSUN: NOL1/NOP2/sun
DNMT2: DNA methyltransferase 2
ALKBH1: 1Alpha-ketoglutarate-dependent dioxygenase ABH1
TET: Ten-eleven translator family proteins
QSOX1: Quiescin sulfhydryl oxidase 1
PVDF: Polyvinylidene fluoride
m6A: N6-methyladenosine
ELISA: Enzyme-linked immunosorbent assay
MS: Mass Spectrometry
1D: One-dimensional
2D: Two-dimensional
TLC: Thin layer chromatography
LC-MS/MS: Liquid chromatography-tandem mass spectrometry
TP53: Tumor protein p53
mTOR: Mammalian target of rapamycin
CTD: C-terminal domain
CSD: Cold shock domain
mRNP: Messenger ribonucleoprotein
MAPK: Mitogen-activated protein kinase
ROS: Reactive Oxygen Species
PAF1: RNA polymerase-associated factor 1
PD-L1: Programmed cell death 1 ligand 1
EMT: Epithelial-mesenchymal transition
SR: Serine/arginine-rich
RRM: RNA recognition motif
HCC: Hepatocellular carcinoma
FAM210A: Family with sequence similarity 210 member A
STAT3: Signal transducer and activator of transcription 3
GSK3β: Glycogen synthase kinase 3 beta Gene
CBX3: Chromobox 3
TGF-β: Transforming Growth Factor Beta
IL-18: Interleukin 18
YAP: Yes1 Associated Transcriptional Regulator
NANOG: Nanog Homeobox
SOX2: SRY-Box Transcription Factor 2
PI3K: Phosphoinositide-3-Kinase
AKT: AKT Serine/Threonine Kinase
LINC00941: Long Intergenic Non-Protein Coding RNA 941
BRCA1: BRCA1 DNA Repair Associated
BRCA2: BRCA2 DNA Repair Associated
VEGFA: Vascular endothelial growth factor A
NSCLC: Non-small cell lung cancer
CDC25a: Cell Division Cycle 25A
HOXC8: Homeobox C8
MMP2: Matrix Metallopeptidase 2
MUC5AC: Mucin 5AC, Oligomeric Mucus/Gel-Forming
MUC1: Mucin 1, Cell Surface Associated
AURKA: Aurora Kinase A
PKM2: Pyruvate Kinase M2
MYC: MYC Proto-Oncogene, BHLH Transcription Factor
HIF1-α: Hypoxia Inducible Factor 1 Subunit Alpha
PRMT5: Protein Arginine Methyltransferase 5
NF-κB: Nuclear Factor Kappa B
RIPK1: Receptor Interacting Serine/Threonine Kinase 1
FOXC2: Forkhead Box C2
VEGF165: Vascular Endothelial Growth Factor 165
E2F1: E2F Transcription Factor 1
SAM: S-adenosyl methionine
f5 C: 5-Formylcytosince
hm5 C: 5-Hydroxymethylcytosine
ca5C: 5-Carboxycytosine.
